# Pioneering In Situ Recrystallization during Bead Milling: A Top-down Approach to Prepare Zeolite A Nanocrystals

**DOI:** 10.1038/srep29210

**Published:** 2016-07-05

**Authors:** Chokkalingam Anand, Yudai Yamaguchi, Zhendong Liu, Sayoko Ibe, Shanmugam P. Elangovan, Toshihiro Ishii, Tsuyoshi Ishikawa, Akira Endo, Tatsuya Okubo, Toru Wakihara

**Affiliations:** 1 The University of Tokyo, Department of Chemical System Engineering, Tokyo, 113-8656, Japan; 2 Ashizawa Finetech Ltd., Akanehama 1-4-2, Narashino, Chiba, 275-8572, Japan; 3 National Institute of Advanced Industrial Science and Technology (AIST), Ibaraki 305-8565, Japan

## Abstract

Top-down approach has been viewed as an efficient and straightforward method to prepare nanosized zeolites. Yet, the mechanical breaking of zeolite causes amorphization, which usually requires a post-milling recrystallization to obtain fully crystalline nanoparticles. Herein we present a facile methodology to prepare zeolite nanocrystals, where milling and recrystallization can be performed *in situ*. A milling apparatus specially designed to work under conditions of high alkalinity and temperature enables the *in situ* recrystallization during milling. Taking zeolite A as an example, we demonstrate its size reduction from ~3 μm to 66 nm in 30 min, which is quite faster than previous methods reported. Three functions, *viz*., miniaturization, amorphization and recrystallization were found to take effect concurrently during this one-pot process. The dynamic balance between these three functions was achieved by adjusting the milling period and temperature, which lead to the tuning of zeolite A particle size. Particle size and crystallinity of the zeolite A nanocrystals were confirmed by X-ray diffraction, scanning electron microscopy, transmission electron microscopy and water adsorption-desorption. This work presents a pioneering advancement in this field of nanosized zeolites, and will facilitate the mass production as well as boost the wide applications of nanosized zeolites.

Synthetic zeolites are important candidates for a wide range of industrial applications. They offer unique structural and textural features that can be further modified for specific needs. Recently, numerous techniques have been reported for the effective improvement of these features. Despite numerous research articles and patents dedicated to exploring the various aspects of zeolite synthesis and applications, interests in these areas seem ever expanding, attracting scientists from interdisciplinary fields^1^,^2^,^3^,^4^,^5^,^6^,^7^,^8^,^9^,^10^. However, an important area of zeolite research, namely the synthesis of zeolite nanocrystals, has not been tapped to its maximum potential^5^,^6^,^7^,^8^,^9^,^10^,^11^,^12^,^13^.

Compared with their micrometer sized counterparts, zeolite nanocrystals have several unique properties which are related to particle-size-induced diffusional freedom and are essential in the fields of catalysis and separation^8^,^9^,^10^. Owing to their fascinating size variations, zeolite nanocrystals are suitable components for designing environmentally benign and economically viable materials of commercial importance^5^,^6^,^7^,^8^,^9^,^10^,^11^,^12^,^13^. To date, several methods have been developed for the preparation of zeolite nanocrystals, such as template-assisted, colloidal solution, ionothermal, dry gel conversion, microwave, sonochemical, and multistep hydrothermal syntheses. Usually, these methods can be categorized into two approaches: bottom-up and top-down^10^,^11^,^12^,^13^,^14^,^15^,^16^,^17^,^18^. The top-down approach, which forms the core of this study, is highly versatile and does not require the complicated procedures used in bottom-up methods.

In our previous studies, zeolite nanocrystals were synthesized by a two-step, top-down strategy. First, zeolite was milled using zirconia beads of size 300 μm. The following recrystallization of milled zeolite with dilute aluminosilicate or silicate solutions then led to nanocrystals with high crystallinity. Zeolite A and ZSM-5, exhibiting excellent ion-exchange and catalytic activity, have been successfully prepared by this technique^17^,^18^,^19^. More importantly, a simplification to achieve a higher yield of zeolite A nanocrystals per batch was also introduced, wherein the aluminosilicate solution was replaced with aqueous NaOH^19^. Similarly, an ultrafast version of this top-down approach using tubular reactors to prepare SSZ-13 and AlPO_4_-5 nanocrystals within a few minutes has been realized^20^.

Inspired by our previous studies, we report a novel yet simple approach for the production of zeolite A nanocrystals that uses a three-integrated factors viz., amorphization, miniaturization and recrystallization. The approach involves an improved method for the reduction of particle size using *in situ* recrystallization during bead milling with aqueous NaOH. This method represents a substantial improvement to our previously reported approach of bead milling followed by post-milling recrystallization^17^,^18^,^19^,^20^. Zirconia beads with a diameter of 500 μm were used to inflict partial structural collapse on zeolite A particles inside an apparatus specially designed to operate under conditions of high alkalinity (pH ~14) and different temperatures (30–60 °C). The thermal stimulation within the milling medium and its alkaline nature simultaneously trigger the recrystallization process, which shapes the milled zeolite A particles into high crystallinity nanocrystals. Since the entire process of *in situ* recrystallization during bead milling takes merely 30 min, it is more attractive for large-scale production than its predecessors (Table S1, Supporting Information).

Three series of zeolite A nanocrystals, prepared using various temperatures and milling times, have been explored to determine the reliability of the *in situ* recrystallization method. The two general nomenclatures *x*°C_*y*min and *x*°C_*y*min_2 h to differentiate samples are based on the milling temperature, time and additional external recrystallization for 2 h when applied (Methods, Supporting Information) respectively. Table S2 shows the list of studied samples and their crystallinity, average particle size and yield. Note that the percentage crystallinity of all samples was calculated by comparing their respective X-ray diffraction (XRD) peak areas with that of raw zeolite A (Equation S1, Supporting Information), and the average particle size was estimated using scanning electron microscopy (SEM) images. The yield of the final products was calculated using Equation S2 (Supporting Information). A schematic representation of the *in situ* recrystallization during bead milling is depicted in Figure S1.

## Results and Discussions

**Figure 1:**
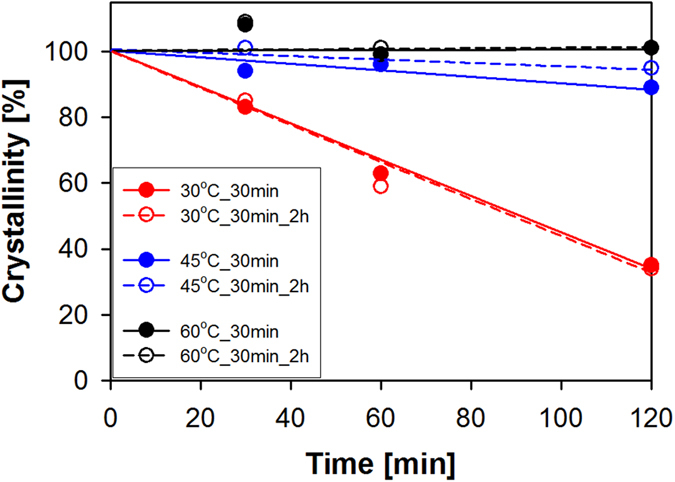
Influence of temperature and external recrystallization on the crystallinity of x°C_30 min and x°C_30 min_2 h samples.

Figure 1 shows the influence of temperature and external recrystallization on the crystallinity of the *x*°C_30 min samples under investigation. At a processing temperature of 30 °C, the crystallinity of the samples was considerably low, indicating that the *in situ* recrystallization is neither proceeding nor noticeably faster at low temperature. This result is consistent with the previous studies because the mechanical force due to bead milling crushed the zeolite A crystals into small pieces but also caused amorphization. In an attempt to restore the crystallinity, an external recrystallization performed outside of the milling apparatus was also conducted using the same alkaline medium and temperature. However, no significant improvement of crystallinity was obtained at 30 °C, even though an external recrystallization time of 2 h was employed, indicating that at low temperature recrystallization proceeds at an unnoticeably slower rate. However, an increase in crystallinity was observed as the temperature was increased from 30 °C to 45 °C and to 60 °C. This result demonstrates that the rate of *in situ* recrystallization is indeed faster at elevated temperatures. For comparison, the external recrystallization was also performed on the 45 °C_30 min and 60 °C_30 min samples, which generated samples named 45 °C_30 min_2 h and 60 °C_30 min_2 h samples, respectively. As observed in Fig. 1, almost no improvement in crystallinity was observed for the 45 °C_30 min_2 h and 60 °C_30 min_2 h samples, probably because the *in situ* recrystallization at these temperatures was adequate to reconstruct the damage caused by bead milling.

As a first step towards optimizing the *in situ* recrystallization during bead milling, we studied the influence of milling time by increasing it from 30 to 120 min at 30 °C. Figures S2 and S3 show the powder XRD patterns and SEM images of the 30 °C_*y*min and 30 °C_*y*min_2 h samples. Evaluation of the XRD peaks reveals a drastic decrease in crystallinity from 83% to 35%, and SEM images show a decrease in particle size from 143 nm to 117 nm (Table S2). Increasing the time from 30 to 120 min causes the formation of higher fractions of non-crystalline portion *i.e*. lower crystallinity in the 30 °C_*y*min samples (Figure S1a and Table S2). Furthermore, the set temperature, *i.e*. 30 °C, seems extremely low for promoting the rate of *in situ* recrystallization. Thus, it was only possible to produce zeolite A nanocrystals with low crystallinity when the time was increased. This result is consistent with that of the 30 °C_*y*min_2 h samples, wherein the crystallinity (Figure S2a) and particle size (Figure S3) remain essentially unchanged even after 2 h external recrystallization (Table S2). Since the process of recrystallization was apparently slower at 30 °C despite increasing the time from 30 to 120 min, the same set of experiments was conducted at elevated temperatures (45 and 60 °C). Thereby, the individual and combined roles of temperature and milling time in promoting the *in situ* recrystallization can be clarified.

**Figure 2:**
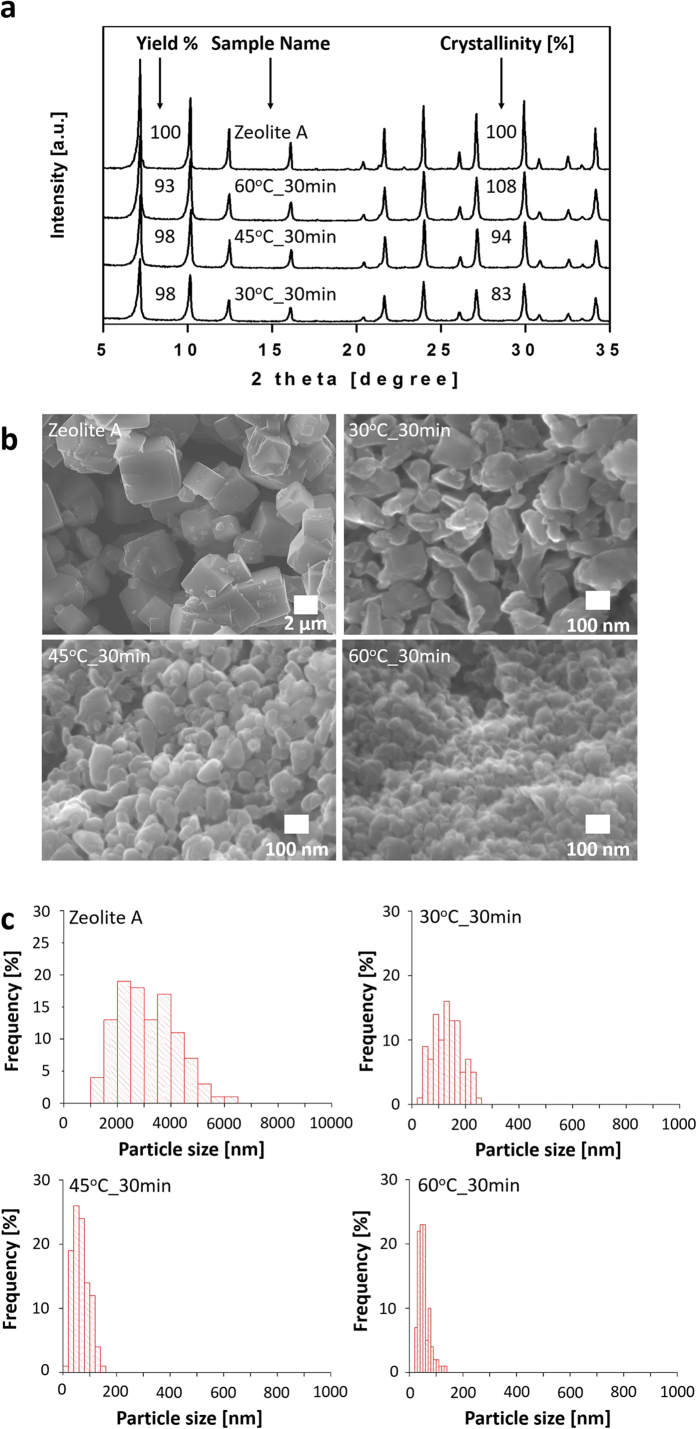
Influence of temperature on the crystallinity of x°C_30 min samples: (**a**) powder XRD patterns. (**b**) SEM images. (**c**) particle size distribution histograms.

Figure 2a,b show the powder XRD patterns and SEM images of *x*°C_30 min samples prepared at different temperatures. A substantial increase in crystallinity from 83% to 108% was achieved by increasing the temperature from 30 to 60 °C (Fig. 2a). Increasing the temperature also decreased the particle size of zeolite A nanocrystals, as evidenced through the SEM images in Fig. 2b. Figure 2c shows the variations in particle size distribution calculated from SEM images of the *x*°C_30 min samples. Though particle size analyzer is considered a specialized technique for measuring particle size, we chose SEM, because of the agglomeration in our samples . The process of recovering the zeolite A nanocrystals from the slurry by washing and drying has led to the capillary force and to the interparticle interactions such as the electrostatic one and the condensation of silanol groups, which resulted in agglomeration. Agglomeration is expected to interfere with the size distribution data, if analysed using particle size analyzer. In order to study the extent of agglomeration, 60 °C_30 min sample was characterized using particle size analyzer over a Shimadzu SALD-7500 and the results are shown in Figure S4 (supporting information). As expected the agglomeration interfered with the particle size data of the 60 °C_30 min sample (Figure S4a). Therefore, prior to particle size analysis, jet milling was used to disperse the agglomerated zeolite A nanocrystals of 60 °C_30 min sample. Figure S4b shows the particle size data of the 60 °C_30 min sample that was dispersed under jet milling, which is quite different from Figure S4a. Even after jet milling the agglomeration can still be found from Figure S4b, which indicates that SEM is suitable for obtaining particle size data in samples with agglomeration.

Apart from higher crystallinity and smaller particle size, the increase in temperature led to highly uniform particle size distribution. At 30 °C, complete control over particle size was difficult to achieve as particles ranging from 60 to 500 nm were produced. However, increasing the temperature to 60 °C enabled production of a uniform size distribution with an average of 66 nm. Note that downsizing zeolite A to highly crystalline particles of uniform size required only 30 min of *in situ* recrystallization during bead milling, which is noticeably faster than its predecessors shown in Table S1 (Supporting Information). The influences of time and external recrystallization on the crystallinity, particle size and yield of the 45 °C_*y*min and 60 °C_*y*min samples were tested and their results are summarized in Figures S5, S6, S7 and S8 and Table S2 (Supporting Information). Unlike the 30 °C_*y*min samples, the decrease in the crystallinity of the 45 °C_*y*min and 60 °C_*y*min samples caused by time variation is relatively small. Furthermore, they exhibited relatively enhanced particle size and crystallinity.

**Figure 3:**
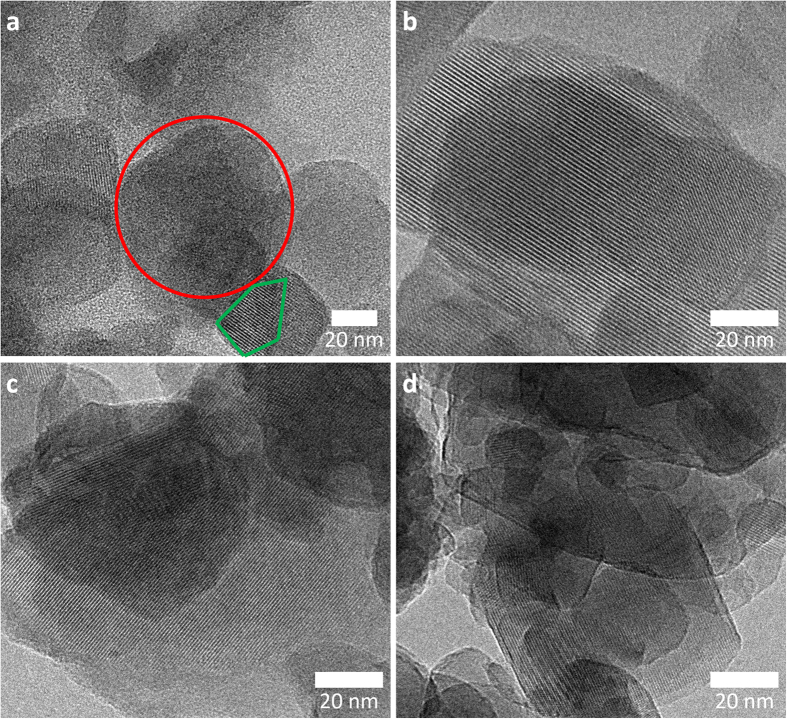
TEM images: (**a**) 30 min milled zeolite A (*i.e*. zeolite A beadmilled without the *in situ* recrystallization; non-crystalline and crystalline particles are indicated within the red and green areas, respectively). (**b**) 30 °C_30 min; (**c**), 45 °C_30 min; (**d**) 60 °C_30 min.

**Figure 4:**
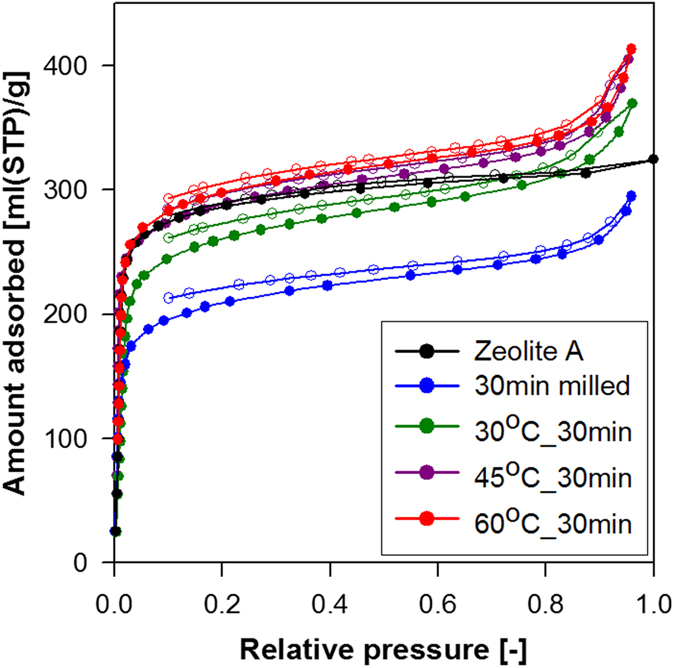
Water vapour adsorption–desorption isotherms of raw, 30 min milled (*i.e*. zeolite A bead milled without *in situ* recrystallization) and *x*°C_30 min zeolite A samples (‘⦁’ indicates adsorption branch of isotherm and ‘⚬’desorption branch of isotherm).

The results indicate that at elevated temperatures, the mobility of the aluminosilicate species in the aqueous NaOH solution is more pronounced, leading to faster recrystallization and faster particle-size reduction as well as narrower size distribution. The extent of damage to the zeolite A lattice was evaluated by comparing the transmission electron microscopy (TEM) image of a sample milled for 30 min without *in situ* recrystallization with those of the *x*°C_30 min samples (Fig. 3). Figure 3a clearly shows the occurrence of non-crystalline zeolite A particles in response to 30 min of bead milling, whereas Fig. 3b,c,d show the highly crystalline array of micropores produced from the damaged zeolite A particles as a result of *in situ* recrystallization during bead milling. The water vapour adsorption–desorption isotherms of the raw, bead milled, *i.e*. zeolite A bead milled without the *in situ* recrystallization, and *x*°C_30 min samples are shown in Fig. 4.

All samples exhibited type I isotherms characteristic of water vapour adsorption onto hydrophilic zeolite A micropores. The variation in the amount of water vapour adsorbed onto the analyzed samples reflects the differences in the degrees of their crystallinity and the particle size. In particular, the amount adsorbed by the 30 min milled sample (*i.e*. the sample bead milled without the *in situ* recrystallization) is substantially less than that adsorbed by the raw and x°C_30 min samples because of the partial loss of crystallinity caused by the milling treatment. The amount of water adsorbed by x°C_30 min samples at high relative pressures increased with temperature, indicating that water molecules adsorbed onto external surfaces and condensed in the interparticle voids of the nanosized samples. These results are in good agreement with the TEM results and demonstrate that increasing the temperature from 30 to 60 °C allows the particle size and crystallinity of zeolite A nanocrystals treated for 30 min to be tuned. The potential of *in situ* recrystallization during bead milling for the faster production of zeolite A nanocrystals has been compared with other established methods^13^,^21^,^22^,^23^ (Table S1, Supporting Information). Table S1 confirms that the formation of zeolite A nanocrystals by our method is the fastest. Our method also shows better yield and more uniform particle size than the other techniques.

The mechanism observed for our top-down *in situ* recrystallization is somehow expected to be different from the bottom-up zeolite crystallization reported by Valtchev *et al*. In their bottom-up approach, Valtchev *et al*. carefully studied the crystallization kinetics under ambient temperature conditions, and proposed a mechanism for bottom-up formation, which views that the nucleation is initiated by an extensive exchange of species between solid and liquid phases during the induction period, and then crystal growth follows by an Ostwald ripening of the crystals^24^,^25^. However, in our opinion and from previous post-milling experiments, we postulate the following mechanism for the *in situ* recrystallization during bead milling. Prior to the milling, raw zeolite A is dispersed in 2M NaOH solution by agitation for 20 min. During this period the zeolite A slightly dissolves to form the interactive species; that is aluminosilicate solution. The resulting aluminosilicate solution is expected to be in a state of equilibrium for the *in situ* recrystallization under bead milling at suitable temperatures. During the bead milling treatment a large number of crystallites were formed. The crystallites act as potential seeds for the *in situ* recrystallization, resulting in zeolite A nanocrystals with high crystallinity. However, the decrease in crystallinity of the 30 °C_*y*min samples despites extending the bead milling time from 30 to 120 min, indicates that at lower temperature milling is more predominant than crystal growth (Table S2 and Figure S2). In this regard, an *ex situ* post-milling recrystallization was performed at 30 °C using 30 min milled zeolite A powder for extended period of time with 2M NaOH. Figure S9 shows the crystallinity of the 30 min milled sample at 30 °C as a function of time. As can be seen from Figure S9, nearly 24 h of post-milling recrystallization at 30 °C is required for achieving 100% crystallinity for the 30 min milled sample. The result confirmed that the *in situ* recrystallization is in fact proceeding at 30 °C; however, its rate is unnoticeably slow as compared to the dominant milling process. Therefore, elevating the temperature from 30 to 60 °C contributes to the higher mobility of aluminosilicate species which greatly improves the rate of *in situ* recrystallization. These results suggest that our *in situ* recrystallization mechanism is kinetically driven to a larger extent.

## Conclusions

In summary, we have developed a strategy for producing zeolite A nanocrystals in a very short period of time, which involves *in situ* recrystallization during bead milling. Since control over the particle size and crystallinity of zeolite A nanocrystals was established by simply adjusting the temperature, the whole process seems to be kinetically driven. Under the right set of condtions (*i.e*. 30 min at 60 °C), *in situ* recrystallization during bead milling holds great potential for the faster preparation of zeolite A nanocrystals with high crystallinity. This facile methodology can serve as a new platform to prepare zeolite nanocrystals that might address issues cutting across a wide range of disciplines, such as catalysis, semiconductors, and drug delivery.

## Experimental Section

### *In situ* recrystallization during bead milling

In a typical milling experiment, commercial zeolite A (4A, LTA-type zeolite, Si/Al = 1.0, cation: Na^+^, Tosoh Co., Japan) was ground inside a milling apparatus (modified LMZ015 Ashizawa Finetech Ltd., Tokyo, Japan with an internal volume of 0.15 L) specially equipped to operate under conditions of high alkalinity (pH ~ 14) and packed with 485 g of ZrO_2_ beads of diameter 500 μm; the mill was operated at a rotation speed of 3000 rpm. Prior to milling, 50 g of zeolite A was dispersed in 500 mL of 2M NaOH solution. Each experiment was performed for 2 h and samples were collected at regular intervals of 30 min, 60 min, and 120 min. The experiments were repeated at various different temperatures (30 °C, 45 °C, and 60 °C).

## Additional Information

**How to cite this article**: Anand, C. *et al*. Pioneering In Situ Recrystallization during Bead Milling: A Top-down Approach to Prepare Zeolite A Nanocrystals. *Sci. Rep*. **6**, 29210; doi: 10.1038/srep29210 (2016).

## Supplementary Material

Supplementary Information
