# Supplementary material for: Pioneering In Situ Recrystallization during Bead Milling: A Top-down Approach to Prepare Zeolite A Nanocrystals
Source: Sci Rep. 2016 Jul 5;6:29210. doi: 10.1038/srep29210 (PMC4932601; doi:10.1038/srep29210)
Supplement: Supplementary Information [file srep29210-s1.pdf]

Supporting Information

Pioneering In Situ Recrystallization during Bead Milling: A Top-down Approach to Prepare Zeolite A Nanocrystals

Chokkalingam Anand[1], Yudai Yamaguchi[1], Zhendong Liu[1], Sayoko Ibe[1], Shanmugam P. Elangovan[1], Toshihiro Ishii[2], Tsuyoshi Ishikawa[2], Akira Endo[3], Tatsuya Okubo[1], and Toru Wakihara[1,*]

[1]The University of Tokyo, Department of Chemical System Engineering, Tokyo, 113-8656, Japan

[2] Ashizawa Finetech Ltd., Akanehama 1-4-2, Narashino, Chiba, 275-8572, Japan

[3] National Institute of Advanced Industrial Science and Technology (AIST), Ibaraki 305-8565, Japan

[*]wakihara@chemsys.t.u-tokyo.ac.jp

Contents

1. Materials and Methods

1.1 Materials

1.2 Characterizations

1.3 Equations

1.4 Methods

2. Supplementary Tables

Table S1 Comparison of various established techniques with in situ recrystallization methodology.

Table S2 Influence of temperature, time and external recrystallization on crystallinity, average particle size, and yield of zeolite A samples prepared by in situ recrystallization during bead milling.

3. Supplementary Figures

Figure S1 Schematic representation of the in situ recrystallization during bead milling.

Figure S2 Influence of time and external recrystallization on zeolite A particle size

	and crystallinity at 30 °C: a, Powder x-ray diffraction patterns with crystallinity data for raw, 30°C_*y*min and 30°C_*y*min_2h zeolite A samples. b, SEM images of 30°C_*y*min zeolite A samples.
Figure S3	SEM images of 30°C_*y*min_2h zeolite A samples.
Figure S4	Particle size analysis: a, 60 °C_30min. b, jet milled 60 °C_30min
Figure S5	Influence of time on zeolite A particle size and crystallinity at 45 °C: a, Powder x-ray diffraction patterns with crystallinity data for raw and 45°C_*y*min zeolite A samples. b, SEM images of raw and 45°C_*y*min zeolite A samples.
Figure S6	Influence of 2 h external recrystallization on zeolite A particle size and crystallinity of 45°C_*y*min_2h zeolite A samples: a, Powder x-ray diffraction patterns with crystallinity data for raw and 45°C_*y*min_2h zeolite A. b, SEM images of raw and 45°C_*y*min_2h zeolite A samples.
Figure S7	Influence of time on zeolite A particle size and crystallinity at 60 °C: a, Powder x-ray diffraction patterns with crystallinity data for raw and 60°C_*y*min zeolite A samples. b, SEM images of raw and 60°C_*y*min zeolite A samples.
Figure S8	Influence of 2 h external recrystallization on zeolite A particle size and crystallinity of 60°C_*y*min_2h zeolite A samples: a, Powder x-ray diffraction patterns with crystallinity data for raw and 60°C_*y*min_2h zeolite A. b, SEM images of raw and 60°C_*y*min_2h zeolite A samples.
Figure S9	Crystallinity of 30°C_30min sample plotted as a function of ex situ post-milling recrystallization time

1. Materials and Methods

1.1 Materials. Zeolite A was purchased from Tosoh Co., Japan (4A, LTA-type zeolite, Si/Al = 1.0, cation: Na^+) and NaOH was purchased from Wako Pure Chemical Industries, Ltd. All chemicals were used as received without further purification. Deionized water from a Millipore water purification system was used in all experiments.

1.2 Characterization. Powder XRD patterns were collected using a Rigaku Ultima IV diffractometer equipped with a Cu-Kα radiation source (λ = 0.15406 nm, 40 kV, 40 mA). The morphology of the products was observed by field-emission scanning electron microscopy (FE-SEM, Hitachi High-Technologies Corp., SU9000) on a microscope operated at an accelerating voltage of 1 kV. In addition to using SEM, size distribution of the samples was measured by particle size analyzer using Shimadzu SALD-7500. The Lattice structural information was captured by field-emission transmission electron microscopy (FE-TEM, 2100F, JEOL, Tokyo, Japan). To investigate the microporous structure of the samples, their water vapour adsorption–desorption isotherms were recorded at 25°C using a BELSORP-aqua (MicrotracBEL Corp.). The samples were degassed at 200°C for 5 h under vacuum before the adsorption measurements.

1.3 Equations. The crystallinity (α) of the zeolite A nanocrystals was determined using the following equation:

$$\alpha\ (\%) = \frac{\text{peak area } 2\theta\ =\ 21.6°, 24°, 27°, 30° \text{for the zeollite A nanocrystals}}{\text{peak area } 2\theta\ =\ 21.6°, 24°, 27°, 30° \text{for the raw zeolite A}} \times 100 \qquad (S1)$$

We calculated the average particle size from SEM images of samples by measuring the individual sizes of a few hundred particles and taking the average of the same. The yield of the final products were calculated using the following equation:

$$\text{Y}\ (\%) = \frac{\text{X1}}{(\text{X} \times \text{Z1})/Z} \times 100 \qquad (S2)$$

where,
‘Y’ – Yield;
‘X’ – total weight of zeolite present in initial slurry;
‘X1’ – weight of final sample after recovery by washing and drying;
‘Z’ – total weight of the initial slurry;
‘Z1‘ – total weight of withdrawn slurry.

1.4 Methods. This study involved two stages: (i) in situ recrystallization during bead milling and (ii) 2 h external recrystallization after in situ processing. A total of three in situ recrystallization during bead milling experiments were performed in succession by varying the system temperature from 30 to 45 °C, and finally to 60 °C. Each experiment was performed for 2 h. Aliquots were collected at intervals of 30 min, 60 min, and 120 min during each in situ run, leading to a total of nine samples. Each sample was divided into two equal parts: one was processed for sample recovery and the other was subjected to external recrystallization for 2 h at a temperature matching the temperature at which the sample was drawn during the experiment. Thus, a total of 18 samples were obtained from the two aforementioned stages. In the process of recovering from the final slurry, samples were washed thrice with pure water to remove NaOH and dried overnight at 80 °C. Samples from the in situ stage were labelled as *x*°C_*y*min, where *x* denotes the in situ system temperature in °C and *y* denotes the in situ system time in min. Similarly, samples from the second stage were denominated *x*°C_*y*min_2h, where *x* and *y* retain their meanings used in the labelling system for the first stage and 2h represents the 2-h period of the external recrystallization performed on the samples after they were collected from the in situ system. For example, 30°C_30min denotes the zeolite A sample recovered directly after the in situ recrystallization during bead milling at 30°C for 30 min, whereas 45°C_60m_2h represents the zeolite A sample first treated by in situ recrystallization during bead milling at 45 °C for 60 min. This is followed by 2 h of external recrystallization at 45 °C after it was collected from the in situ system. Furthermore, an ex situ post-milling recrystallization was performed at 30°C using 30 min milled zeolite A powder for extended period of time with 2M NaOH.

2. Supplementary Tables

Table S1 Comparison of various established techniques for the preparation of zeolite nanocrystals.

Nanocrystal	Method	Particle size [nm]	Time [h]	Temperature [°C]	Yield [%]
Zeolite A this work	In Situ top-down approach-this work without OSDA[a)]	66	0.5	60	>90
Zeolite A[1)]	Colloidal solution method using OSDA[a)]	40-80	72	22	-
NaY[2)]	Twostage varying temperature method using OSDA[a)]	50	84	95	<10
FAU zeolite (Y10)[3)]	Hydrothermal crystallization method without OSDA[a)]	10-15	45	50	>80
Zeolite A[4)]	Colloidal solutions method without OSDA[a)]	200	72	Room temperature	83

[1)] Reference [1]; [2)] Reference [2]; [3)] Reference [3]; [4)] Reference [4]; [a)] OSDA-organic structure directing agent.

References

1. Mintova, A. S., Olson, N. H., Valtchev, V. & Bein, T. Mechanism of zeolite A nanocrystal growth from colloids at room temperature. *Science* **283,** 958-960 (1999).
2. Song, W., Li, G., Grassian, V. H. & Larsen, S. C. Development of improved materials for environmental applications: nanocrystalline NaY zeolites. *Environ. Sci. Technol.* **39,** 1214-1220 (2005).
3. Awala, H. et al. Template-free nanosized faujasite-type zeolites. *Nat. Mater.* **14,** 447-451 (2015).
4. Valtchev, V., Tosheva, L. & Bozhiolv, K. N. Synthesis of zeolite Nanocrystals at room temperature. *Langmuir* **21,** 10724-10729 (2005).

Table S2 Influence of time, temperature and external recrystallization on the crystallinity, average particle size and yield of zeolite A samples prepared by in situ recrystallization during bead milling.

No.	Samples	Crystallinity[a)] [%] ± 7 [%] error	Average particle size[b)] [nm]	Yield [%] ± 5 [%] error
1	Raw zeolite A	100	3000	100
2	30°C_30min	83	143	98
3	30°C_60min	63	128	98
4	30°C_120min	35	117	98
5	30°C_30min_2h	85	120	101
6	30°C_60min_2h	59	82	100
7	30°C_120min_2h	34	75	100
8	45°C_30min	96	81	98
9	45°C_60min	94	78	100
10	45°C_120min	89	69	100
11	45°C_30min_2h	101	89	107
12	45°C_60min_2h	96	81	106
13	45°C_120min_2h	95	62	102
14	60°C_30min	108	66	93
15	60°C_60min	99	49	95
16	60°C_120min	101	61	98
17	60°C_30min_2h	108	70	95
18	60°C_60min_2h	101	63	97
19	60°C_120min_2h	101	57	101

[a)]Derived from XRD peak fitting results and calculated according to Equation (S1); [b)]Derived from high-magnification SEM images

3. Supplementary Figures

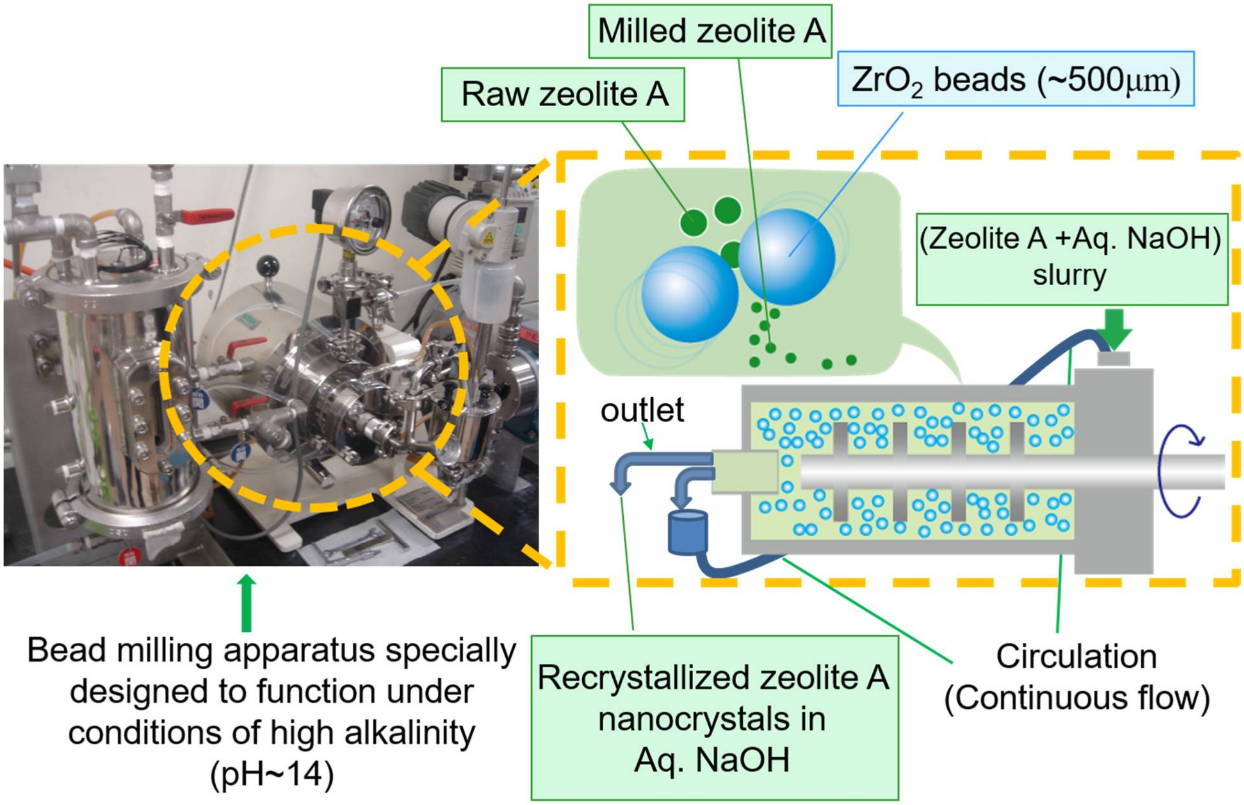

Figure S1. Schematic representation of in situ recrystallization during bead milling.

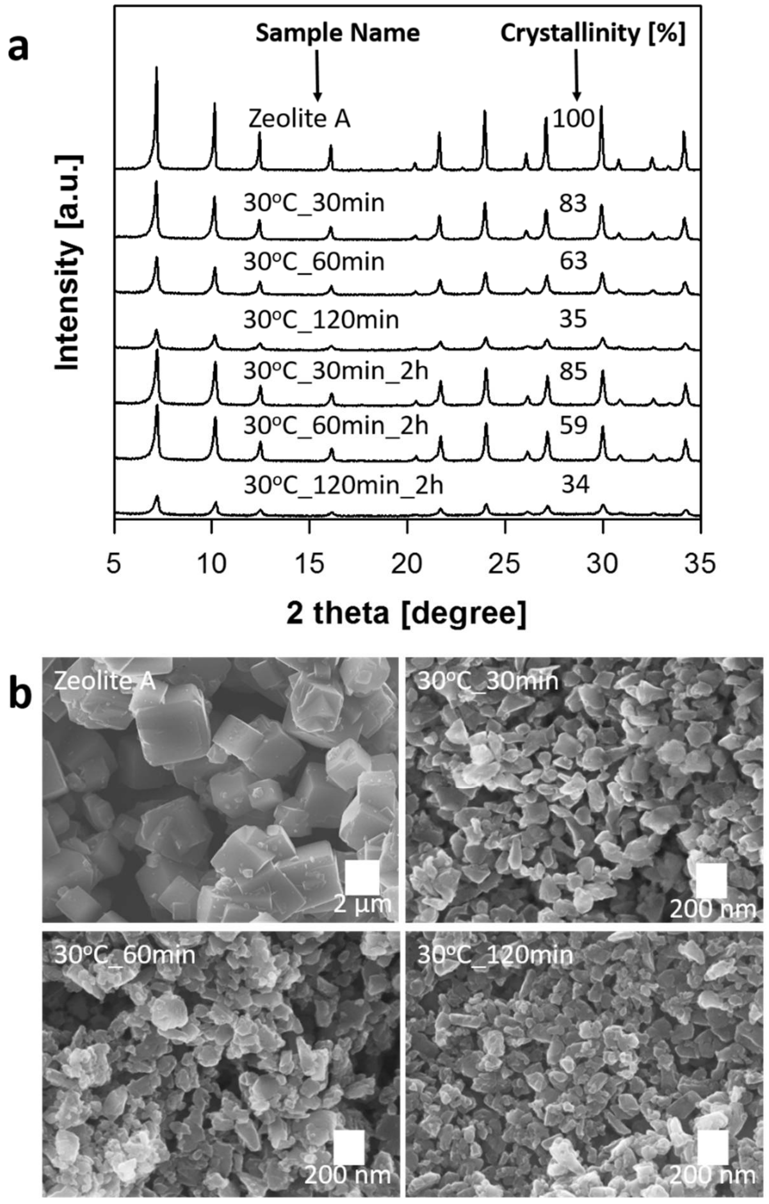

Figure S2. Influence of time and external recrystallization on zeolite A particle size and crystallinity at 30 °C: a, Powder x-ray diffraction patterns with crystallinity data for raw, 30°C_*y*min, and 30°C_*y*min_2h zeolite A samples. b, SEM images of 30°C_*y*min zeolite A samples.

Figure S2a shows a decrease in the crystallinity of the 30°C_*y*min and 30°C_*y*min_2h samples as time is increased from 30 to 120 min. Figure S2b shows the influence of time on the particle size of the 30°C_*y*min samples.

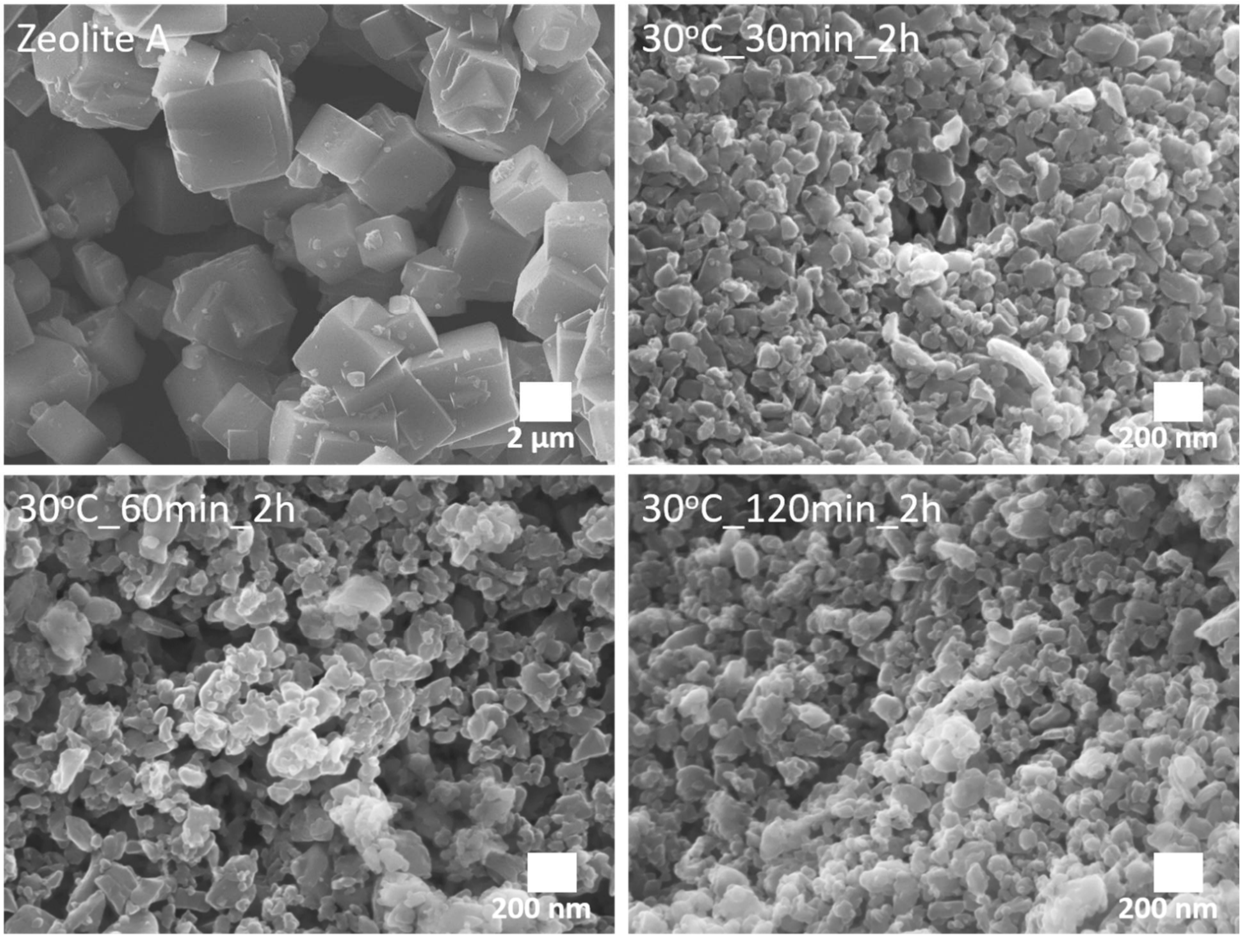

Figure S3. SEM images showing the influence of external recrystallization on the particle size of 30°C_*y*min_2h zeolite A samples.

Figure S3 confirms that the morphology and particle size of the 30°C_*y*min_2h samples are not altered by the external recrystallization; the results for these samples resemble those obtained for the 30°C_*y*min sample series.

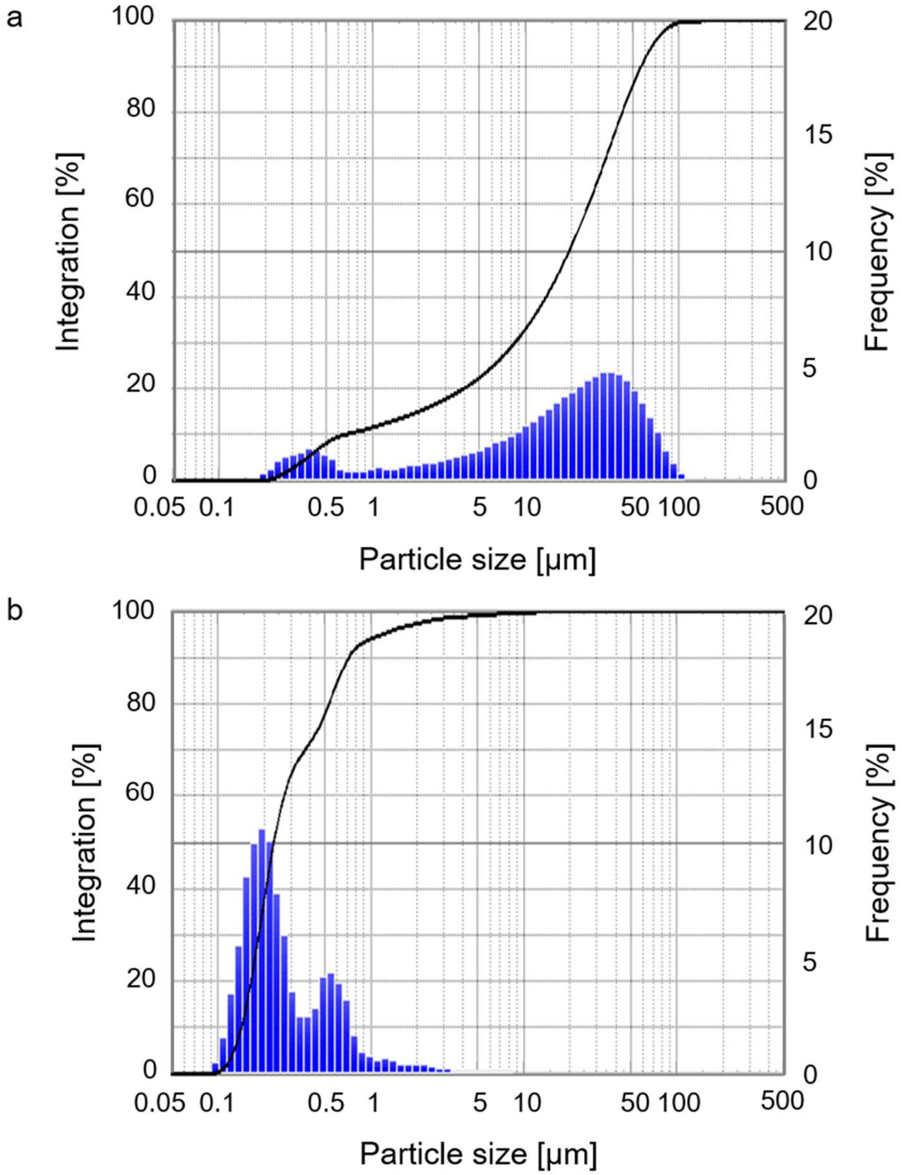

Figure S4. Particle size analysis: a, 60°C_30min. b, jet milled 60°C_30min.

The interference of agglomeration in the particle size data of 60°C_30min sample is observed from Figure S4a, whereas the level of agglomeration in 60°C_30min sample was reduced to a major extent when dispersed with jet milling as observed from Figure S4b.

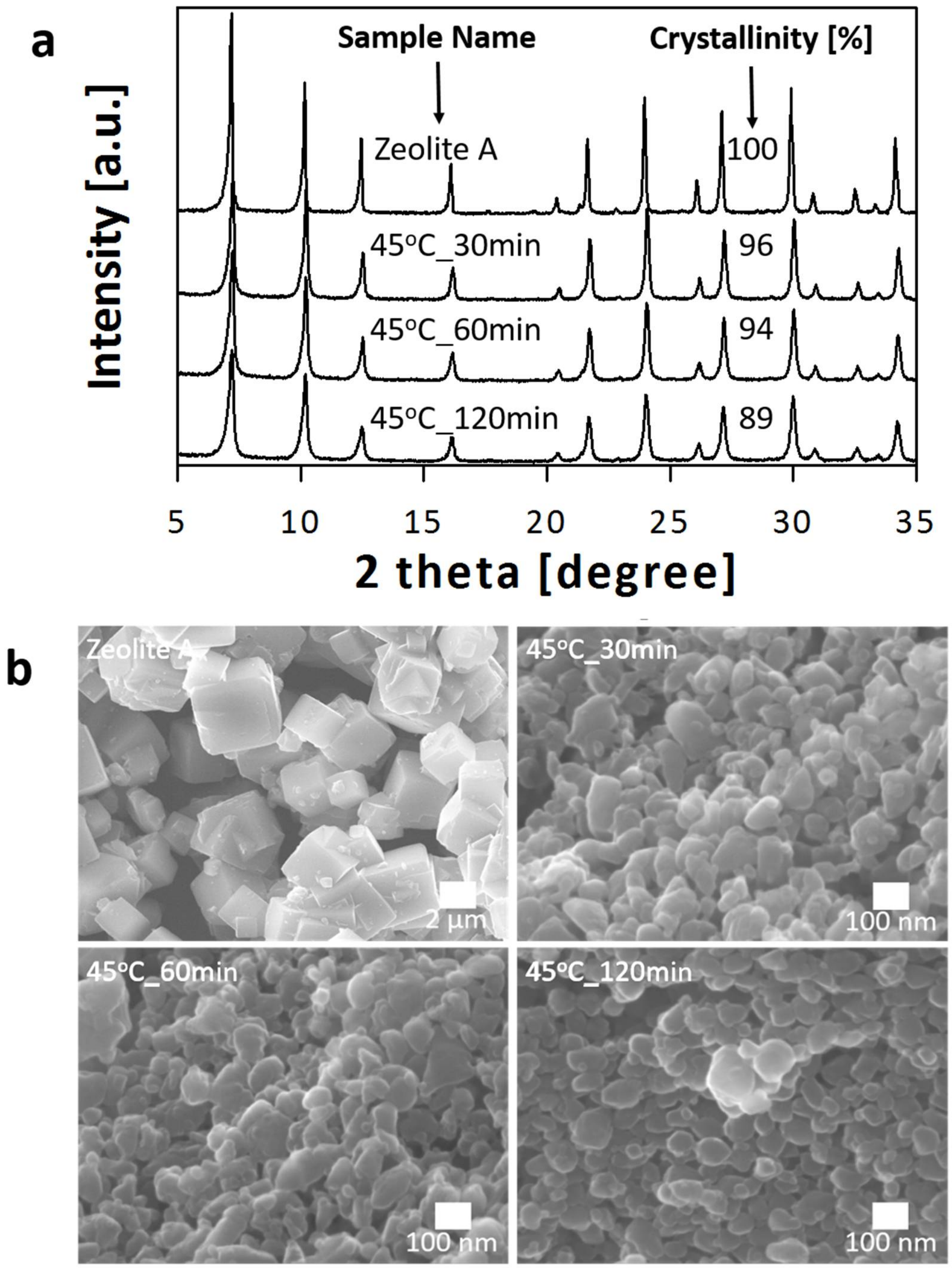

Figure S5. Influence of time on zeolite A particle size and crystallinity at 45 °C: a) Powder x-ray diffraction patterns with crystallinity data for raw and 45°C_*y*min zeolite A samples; b) SEM images of raw and 45°C_*y*min zeolite A samples.

The decreases in crystallinity and particle size of the 45°C_*y*min zeolite A samples as the time was increased from 30 to 120 min are clearly evident in their powder XRD patterns (Figure S4a) and SEM images (Figure S4b), respectively.

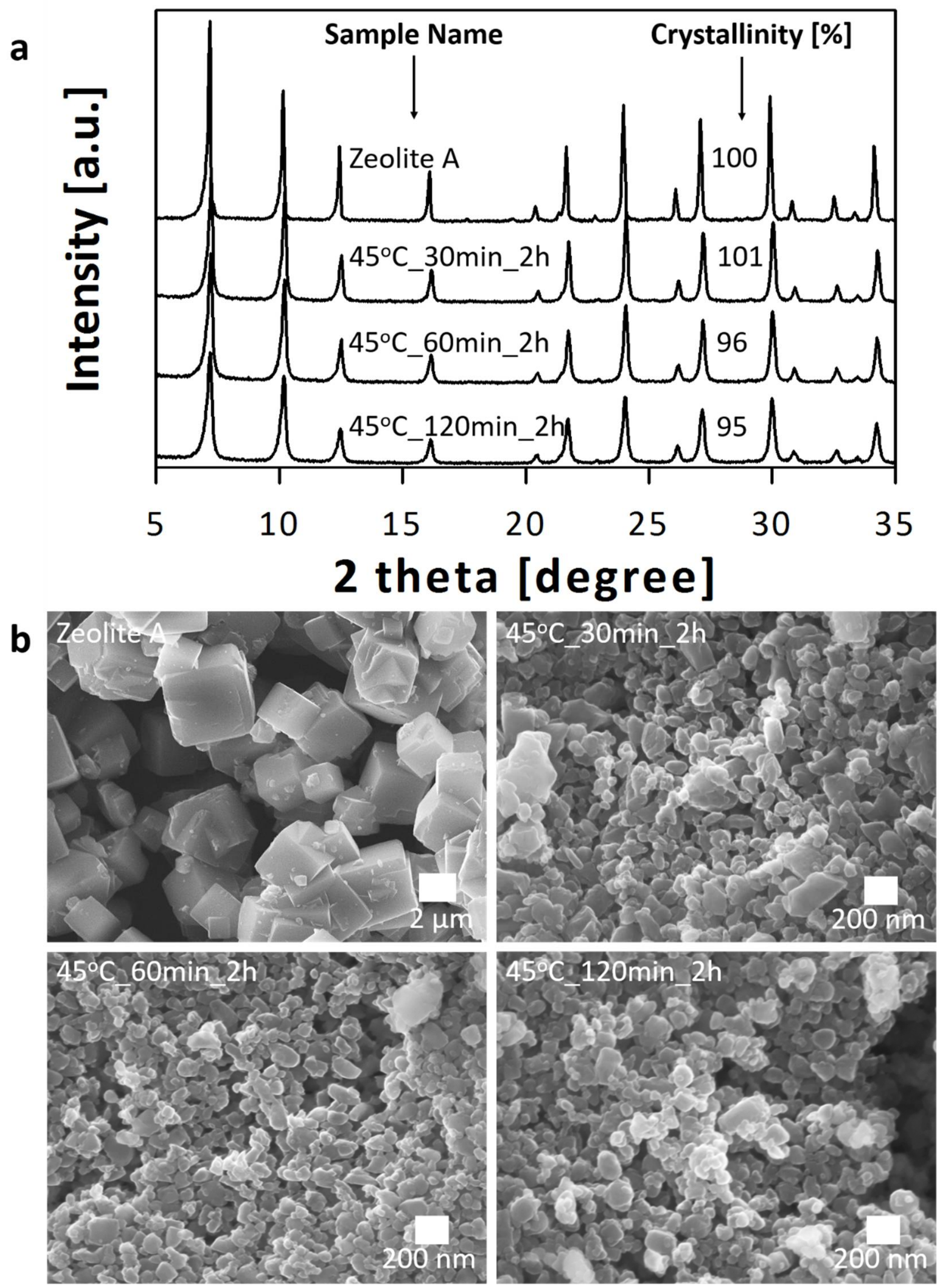

Figure S6. Influence of external recrystallization on the zeolite A particle size and crystallinity of 45°C_*y*min_2h zeolite A samples: a, Powder x-ray diffraction patterns with crystallinity data for raw and 45°C_*y*min_2h zeolite A samples. b, SEM images of raw and 45°C_*y*min_2h zeolite A samples.

The crystallinity (Figure S5a) and particle size (Figure S5b) trends observed for the 45°C_*y*min_2h samples are highly similar to those observed for the 45°C_*y*min samples (Figure S4).

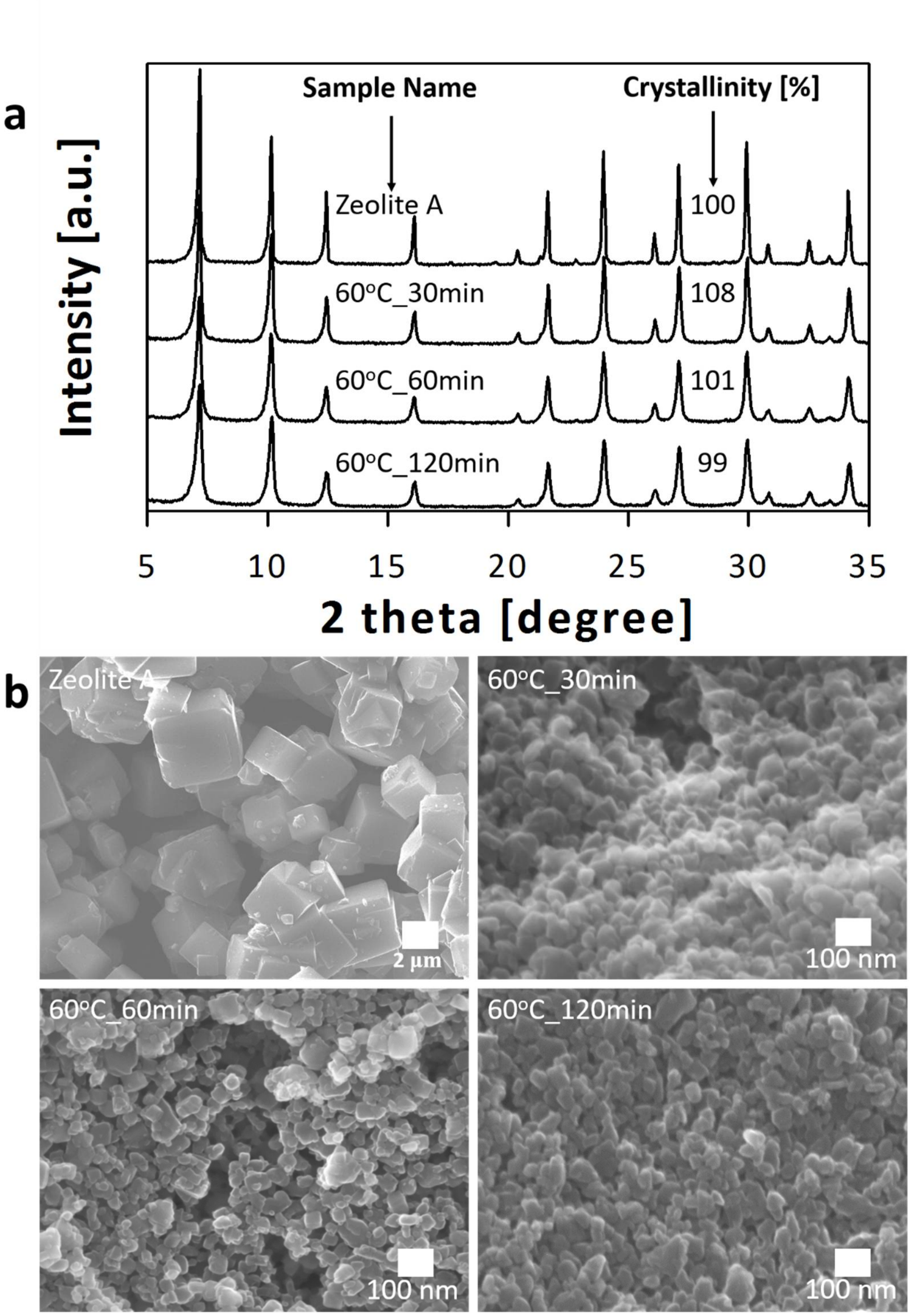

Figure S7. Influence of time on zeolite A particle size and crystallinity at 60 °C: a, Powder x-ray diffraction patterns with crystallinity data for raw and 60°C_*y*min zeolite A samples. b, SEM images of raw and 60°C_*y*min zeolite A samples.

The crystallinity and particle size of the 60°C_*y*min samples decreased as the time was increased from 30 to 120 min, as evidenced from their respective powder XRD patterns (Figure S6a) and SEM images (Figure S6b).

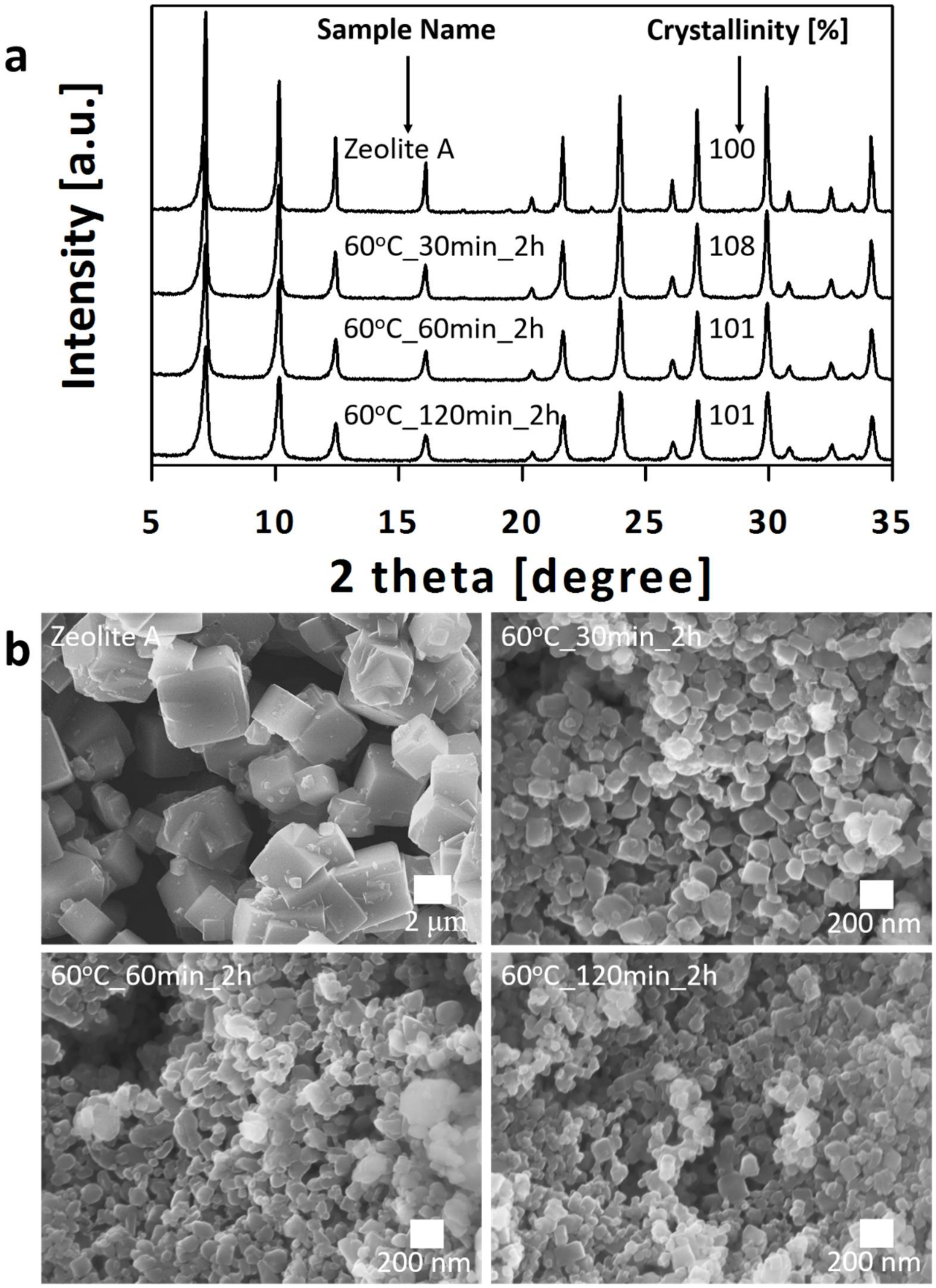

Figure S8. Influence of external recrystallization on the zeolite A particle size and crystallinity of 60°C_*y*min_2h zeolite A samples: a, Powder x-ray diffraction patterns with crystallinity data for raw and 60°C_*y*min_2h zeolite A samples. b, SEM images of raw and 60°C_*y*min_2h zeolite A samples.

Powder XRD patterns (Figure S7a) confirmed that the crystallinity of the 60°C_*y*min_2h samples was not enhanced by the 2 h external recrystallization. Similarly, the particle-size variations remain essentially unchanged even after the external recrystallization (Figure S7b).

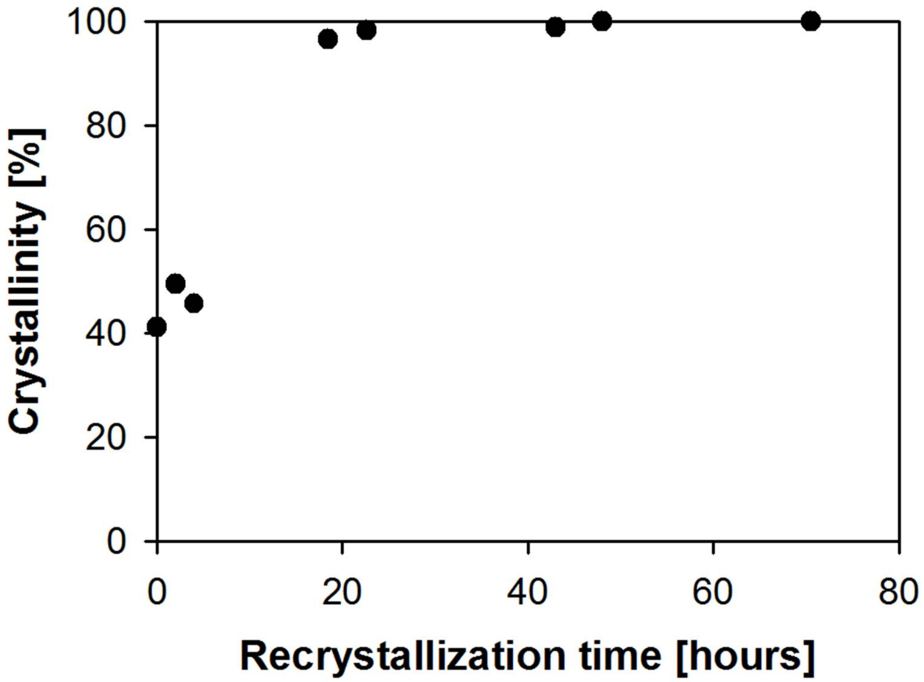

Figure S9. Crystallinity of 30°C_30min sample plotted as a function of ex situ post-milling recrystallization time.

Ex situ post-milling recrystallization was performed at 30°C using 30 min milled zeolite A powder. The crystallinity data of 30 min milled sample obtained from the ex situ post-milling recrystallization at 30 °C as a function of time is shown in Figure S9, which indicates that the in situ recrystallization is in fact proceeding at lower temperature (i.e. 30 °C) but at an unnoticeably slower rate that it gets shielded by the dominant milling process.
